# A novel synthetic flavonoid with potent antibacterial properties: *In vitro* activity and proposed mode of action

**DOI:** 10.1371/journal.pone.0194898

**Published:** 2018-04-04

**Authors:** Cornelia Babii, Gabriela Mihalache, Lucian Gabriel Bahrin, Anca-Narcisa Neagu, Irina Gostin, Cosmin Teodor Mihai, Laura-Gabriela Sârbu, Lucian Mihail Birsa, Marius Stefan

**Affiliations:** 1 Faculty of Biology, Biology Department, The Alexandru Ioan Cuza University of Iasi, Iasi, Romania; 2 Integrated Center for Environmental Sciences Studies - North Eastern, CERNESIM, The Alexandru Ioan Cuza University of Iasi, Iasi, Romania; 3 Faculty of Chemistry, The Alexandru Ioan Cuza University of Iasi, Iasi, Romania; 4 Interdisciplinary Research Department – Science, The Alexandru Ioan Cuza University of Iasi, Iasi, Romania; 5 Advanced Research and Development Center in Experimental Medicine CEMEX, University of Medicine and Pharmacy “Grigore T. Popa” of Iasi, Iași, Romania; Universite Paris-Sud, FRANCE

## Abstract

The emergence of pathogenic multidrug-resistant bacteria demands new approaches in finding effective antibacterial agents. Synthetic flavonoids could be a reliable solution due to their important antimicrobial activity. We report here the potent *in vitro* antibacterial activity of **ClCl-flav**—a novel synthetic tricyclic flavonoid. The antimicrobial effects were tested using the minimum inhibitory concentration (MIC), time kill and biofilm formation assays. Fluorescence microscopy and scanning electron microscopy were employed to study the mechanism of action. MTT test was used to assess the cytotoxicity of **ClCl-flav**. Our results showed that Gram positive bacteria were more sensitive (MIC = 0.24 μg/mL) to **ClCl-flav** compared to the Gram negative ones (MIC = 3.9 μg/mL). We found that our compound showed significantly enhanced antibacterial activities, 32 to 72-fold more active than other synthetic flavonoids. **ClCl-flav** showed bactericidal activity at concentrations ranging from 0.48 to 15.62 μg/mL. At twice the MIC, all *Escherichia coli* and *Klebsiella pneumoniae* cells were killed within 1 h. Also **ClCl-flav** presented good anti-biofilm activity. The mechanism of action is related to the impairment of the cell membrane integrity. No or very low cytotoxicity was evidenced at effective concentrations against Vero cells. Based on the strong antibacterial activity and cytotoxicity assessment, **ClCl-flav** has a good potential for the design of new antimicrobial agents.

## Introduction

The emergence of multidrug-resistant bacteria is causing serious problems in the medical community with widespread health and socio-economic implications. This problem is closely related to the growing public concern regarding the negative effects of antimicrobial drugs on human health [1]. Therefore, there is a huge demand for novel antibacterial agents effective against pathogenic bacteria resistant to the current antibiotics. A reliable solution to this problem could be the use of natural compounds such as flavonoids—a group of heterocyclic organic compounds that occur widely in the plant kingdom. For centuries, flavonoids have been used in the attempt to treat human diseases due to their antibacterial, antifungal, antiviral, anti-allergic, anti-inflammatory and antioxidant activities [2]. Several flavonoids including apigenin, galangin, flavone and flavonol glycosides, isoflavones, flavanones and chalcones have been shown to possess potent antibacterial activity [3–6]. Nowadays, antibacterial research is related more to semisynthetic and synthetic flavonoids due to their higher antimicrobial activity. Some of these compounds were found to be 16–32 folds more active than natural flavonoids [7], as our previous studies have shown as well [8, 9]. Despite the impressive number of studies on flavonoids as antimicrobial agents, their mechanism of action still remains unclear. Our literature survey revealed that their antibacterial activity may be attributed to three mechanisms: cytoplasmic membrane damage [10], inhibition of nucleic acid synthesis [11] and inhibition of energy metabolism [12]. Moreover, recent developments showed that the results of some studies concerning the mechanism of action are not as reliable as first thought, raising doubts on the conclusions drawn so far [7].

In this study, a novel synthetic sulfur containing tricyclic flavonoid with chlorine as halogen substituent at the benzopyran core (**ClCl-flav**) was tested *in vitro* for antibacterial activity against clinically significant microorganisms such as *Staphylococcus aureus*, *Escherichia coli* and *Klebsiella pneumoniae*. Nothing is known so far about the **ClCl-flav** mechanism of action. Since this will have implications on its spectrum of activity and the development of resistance, we examined the mechanism of action of **ClCl-flav** against representative Gram positive and Gram negative bacteria.

## Materials and methods

### Antimicrobial agent and bacterial strains

**Fig 1:**
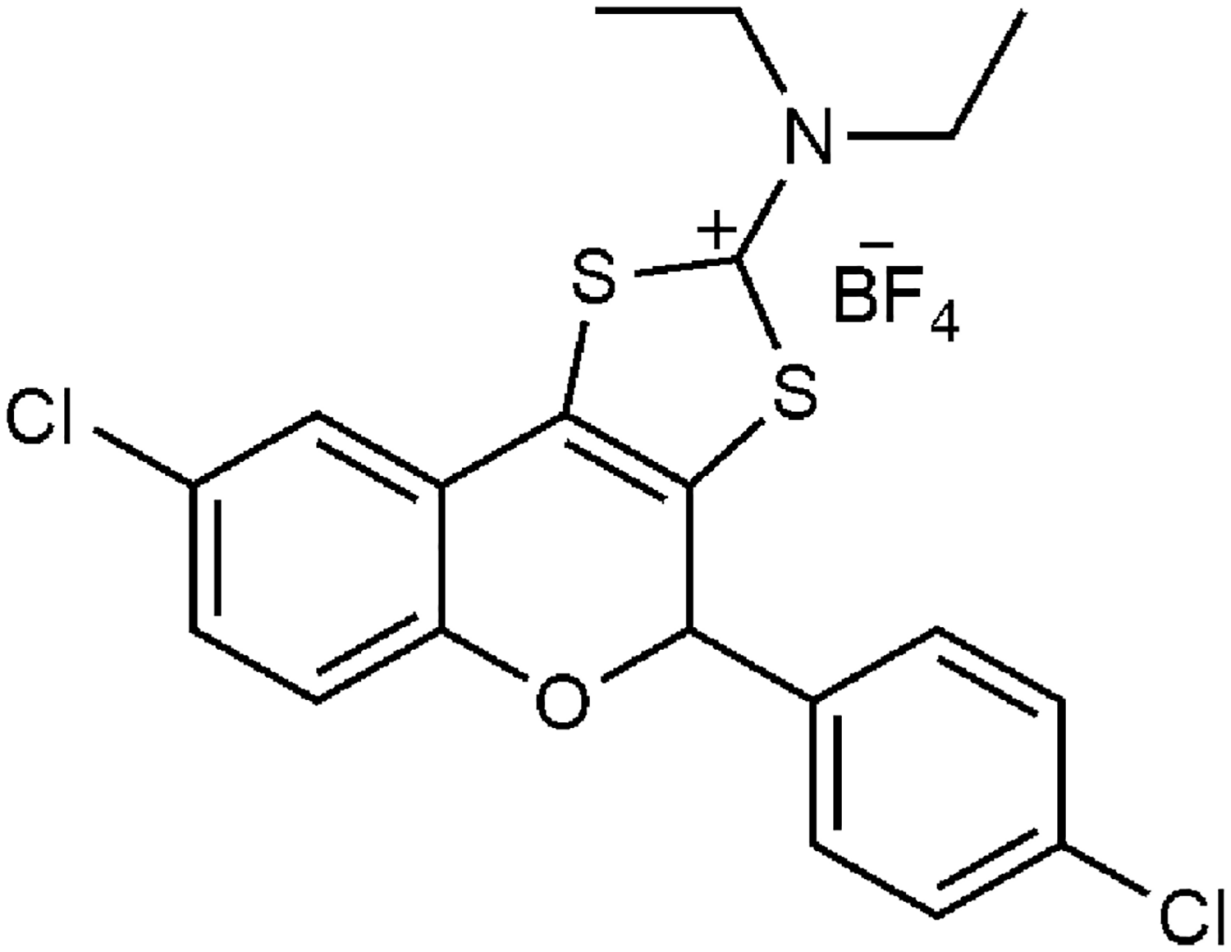
Structure of flavonoid ClCl-flav.

Tricyclic flavonoid **ClCl-flav** (Fig 1) has been synthesized from the corresponding 5-bromo-2-hydroxyphenacyl-*N*,*N*-diethyldithiocarbamate through a two-step reaction sequence. To a mixture of sulfuric acid (0.25 mL) and acetic acid (0.75 mL), the appropriate flavanone (132 mg, 0.3 mM) was added and the resulting solution was heated to 80 °C for 20 min. The reaction mixture was then left to cool down to room temperature and a solution of sodium tetrafluoroborate (110 mg) in water (5 mL) was added dropwise, with vigorous stirring. The precipitate was filtered, washed thoroughly with water and recrystallized from ethanol, yielding the desired product in the form of colorless crystals. The structure and purity (>99%) of the final compound have been established by NMR, MS, IR and elemental analysis [13]. Also the stability of **ClCl-flav** towards Mueller-Hinton broth and phosphate buffer saline has been monitored by UV-Vis spectroscopy. The tricyclic flavonoid proved to be stable as demonstrated by the constant extinction coefficient of the maximum absorption wavelength of **ClCl-flav** over a time span equivalent to the susceptibility tests performed.

*Staphylococcus aureus* ATCC 25923, *Bacillus subtilis* ATCC 6633, *Escherichia coli* ATCC 25922 and *Klebsiella pneumoniae* ATCC 13883 strains were obtained from the culture collection of the Microbiology Laboratory, Alexandru Ioan Cuza University of Iasi and used as test organisms. All strains were stored at -80 °C in 15% glycerol stocks. Before testing, the microorganisms were transferred on Mueller-Hinton agar (MHA, Liofilchem, Italy) and cultured overnight at 37 °C. Subsequently, 10 mL of Mueller Hinton broth (MHB, Scharlau, Spain) were inoculated with one representative colony of each organism taken from MHA, cultured overnight (37 °C, 190 rpm) and used as source of inoculum for each experiment.

### Preparation of bacterial suspensions

Unless stated otherwise, bacterial cells suspensions in the exponential phase of growth were prepared as follows: cultures in MHB obtained as mentioned above were inoculated into 50 mL of the same growth medium and incubated at 37 °C with shaking (190 rpm) until an optical density at 600 nm (OD600) of 0.1 (approximately 1.5 × 10^8^ CFU/mL) was reached. After incubation, the bacteria were separated from the growth medium by centrifugation at 10,000 × g for 15 min at 4 °C, washed twice with phosphate-buffered saline (PBS, pH 7.4) and resuspended in PBS. The OD600 of the cells suspensions were checked every time and adjusted to 0.1.

### Susceptibility determinations

The screening of the antibacterial effective concentrations was performed using a colorimetric broth microdilution technique, as we previously described [8]. A concentration range of **ClCl-flav** between 0.12–250 μg/mL was tested with DMSO as solvent. DMSO was used as control with concentrations ranging from 25 to 0.012% (v/v) and ampicillin and kanamycin as reference antibiotics. The minimum inhibitory concentration (MIC) was considered as the lowest concentration at which bacteria failed to grow in MHB supplemented with **ClCl-flav** after 18 hours, but cultured when plated onto MHA.

#### Bacterial growth analysis

An overnight preculture in MHB was prepared in order to evaluate the bacterial growth in the presence of **ClCl-flav**. A volume of 1 mL from the preculture adjusted to approximately 1.5 × 10^8^ CFU/mL was added in 100 mL MHB medium with ½ MIC, MIC, 2 × MIC values as final concentrations of **ClCl-flav**. Inoculated MHB medium supplemented with DMSO without **ClCl-flav** at appropriate concentrations was used as control. All flasks were incubated on an orbital shaker at 190 rpm at 37 °C for 24 h. Growth rates were determined by measuring OD600 at each hour up to 12 hours and at 24 hours, using a Beckman Coulter DU 730 spectrophotometer.

#### Bacterial killing assay

The killing rate of tested bacteria by **ClCl-flav** was determined by measuring the reduction in the number of colony-forming units (CFU) per mL. Bactericidal activity was defined by 99.9% killing of the final inoculum by noting a ≥ 3-log_10_ decrease in CFU/mL. Bacterial suspensions were prepared as described above with cell densities adjusted to approximately 0.5 McFarland. A volume of 100 μL from bacterial suspensions was added to 10 mL PBS with different concentrations of **ClCl-flav**. Controls were prepared similarly using DMSO at appropriate concentrations. All flasks were incubated for 24 hours at 37 °C. Samples were removed at each hour up to 12 hours and at 24 hours, serially diluted, plated onto MHA and incubated overnight at 37 °C. Colonies were counted after 24 hour incubation and the viable cell number reported as CFU per mL was transformed into log_10_ values [14].

### *In vitro* biofilm formation

Bacterial biofilms were allowed to form in flat-bottomed sterile 96-well microplates with lids (Becton Dickinson) following the method described by Rukayadi *et al*. [3] with some modifications. The wells were filled with 250 mL cell suspension in MHB medium with the final density adjusted to approximately 1 × 10^5^ CFU/mL and incubated at 37 °C without agitation for 24 h. After incubation, the medium was discarded and nonadherent cells were removed by thoroughly washing the biofilm three times with sterile PBS. After plate drying, the same volumes of MHB with DMSO (control) and MHB supplemented with different concentrations of **ClCl-flav** (samples) were added in each well and the plates were incubated at 37 °C without agitation for 24 h.

The quantification of bacterial biofilms was carried out using a method previously described [15]. Briefly, washed adherent cells, as described above, were stained with 1% crystal violet. The dye was solubilized using 30% glacial acetic acid for 15 min. Relative biofilm formation was assayed by reading optical density at 595 nm using a Beckman Coulter DU 730 spectrophotometer.

### Evaluation of membrane integrity

#### Loss of 260 nm absorbing material

Measurement of **ClCl-flav**-induced leakage of 260 nm absorbing compounds was performed following the method described by Carson *et al*. with some changes [14]. Cell suspensions were prepared as described above. Pretreatment samples were taken and filtered using 0.2 μm-pore-size filters (Carl Roth, Germany). **ClCl-flav** was added at final concentrations equivalent to corresponding MICs. Additional samples were removed each hour, up to 5 hours, filtered as described above and the OD260s were determined using a Beckman Coulter DU720 Life Sciences spectrophotometer. Filtrates of the PBS and **ClCl-flav** at appropriate concentrations were used as blanks.

#### Acridine orange/propidium iodide and ethidium bromide uptake

The bacterial cell membrane integrity was assessed also by capturing fluorescence photomicrographs illustrating the uptake of the fluorescent nucleic acid stains acridine orange/propidium iodide (AO/PI—for *S*. *aureus*) and ethidium bromide (EB—for *E*. *coli*), according to a previously described procedure [16, 17]. In order to perform this assay, exponential-phase cells suspensions were prepared as we mentioned above, followed by the addition of **ClCl-flav** (final concentration equal to 2 × MIC values) or DMSO as control. During the 37 °C incubation, samples of treated and untreated cells were taken at 15, 30, 60, 120 and 180 min. Prior to microscopic examination, 20 μL of EB (0.1 mg/mL stock solution) or 30 μL AO/PI (1:1, 0.1 mg/mL respectively 1 mg/mL stock solutions) were added to 200 μL aliquots containing *E*. *coli* or *S*. *aureus* cells respectively, followed by a 10 min incubation time in the dark. The fluorescent cells in the population were counted using a Leica Confocal Laser Scanning Microscope (TCS SPE DM 5500Q) using the I3 blue excitation range filter cube (BP 450–490 nm band pass filter) and the N2.1 green excitation filter cube (BP 515–560 nm band pass filter). At least five random, independent images were captured per sample and the ratio between fluorescent cells and total cells visualized with differential interference contrast (DIC) for *E*. *coli* or ratio between fluorescent cells colored in red and total fluorescent cells for *S*. *aureus* was calculated as percentage.

### Scanning electron microscopy (SEM)

Suspensions in PBS of the logarithmic growth phase bacterial cells were incubated for 6 h with **ClCl-flav** at final concentrations of MIC and 2 × MIC values and DMSO as control. Prior to SEM analysis, samples containing untreated and treated bacterial cells were deposited on a Millipore 0.2 μm filter. The bacterial cells were further fixed in glutaraldehyde 2.5% for 2 h, dehydrated in a stepwise concentration gradient of ethanol solutions (70, 80, 90 and 100%) and acetone. The filters containing bacterial cells were dried with CO_2_ in an EMS 850 critical point dryer, sputter-coated with a 30 nm layer of gold (EMS 550X Sputter Coater) and examined by SEM (Tescan Vega II SBH) at an acceleration voltage of 27.88 kV.

### MTT assay

The cytotoxicity of **ClCl-flav** was assessed using MTT test [18]. HeLa and Vero cells obtained from the culture collection of Alexandru Ioan Cuza University of Iasi were used. Cells were cultivated in Dulbecco’s modified Eagle’s medium using 96 well plates and were allowed to grow overnight in a CO_2_ incubator at 37 °C with 5% CO_2_ and 95% humidified atmosphere. After incubation the medium was discarded and cells washed. A renewal complete medium supplemented with **ClCl-flav** at final concentrations of 0.25, 0.5, 2, 4, 8, 16 and 32 μg/mL was added. Controls were incubated with equivalent concentrations of DMSO. After 24 hours the medium was discarded, the cells were washed with PBS and covered with 100 μL fresh medium. A volume of 10 μL of MTT (5 mg/mL) was added in medium and cells were incubated for 3 hours. DMSO was used to solve the blue crystals of formazan and absorbance was measured at 540 nm.

### Statistical analysis

All experiments were performed in triplicate. The data are presented as mean (n = 3) ± S.E.M. The statistical evaluation of the results was carried out by ANOVA single factor and ANOVA two factors with replication using XLSTAT software package version 7.5.2. Differences between groups were considered significant when P < 0.05.

## Results

We report here the results concerning the **ClCl-flav** antimicrobial activity against some representative Gram positive and Gram negative bacteria, as the synthesis and characterization of **ClCl-flav** were previously described [13].

### Antimicrobial activity

**ClCl-flav** exhibited potent inhibitory activity against all tested bacterial strains, as it is shown in Table 1. However, it should be noted that the Gram positive bacteria were more sensitive (MIC = 0.24 μg/mL) to **ClCl-flav** compared to the Gram negative bacteria (MIC = 3.9 μg/mL).

**Table 1:** Minimum inhibitory concentrations of ClCl-flav against tested bacteria.

Sample	Bacterial strain			
	MIC (μg/mL)			
	*S*. *aureus*	*B*. *subtilis*	*E*. *coli*	*K*. *pneumoniae*
ClCl-flav	**0.24**	**0.24**	**3.9**	**3.9**
Ampicillin	7.81	0.24	7.81	> 250
Kanamycin	1.95	1.95	7.81	1.95
DMSO	250	250	125	125

MIC = minimum inhibitory concentration

#### Effect of ClCl-flav on bacterial growth

**Fig 2:**
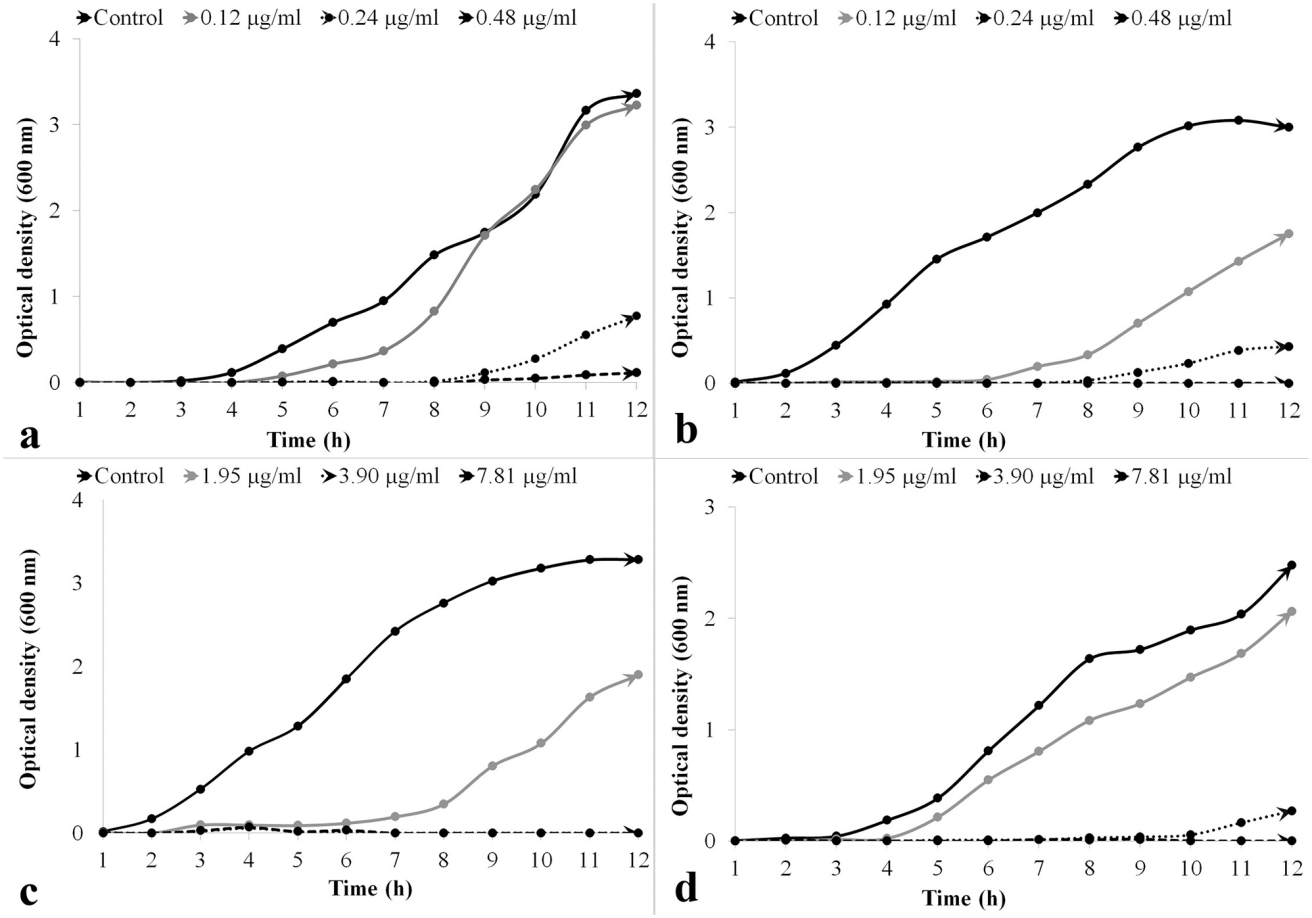
Effect of ClCl-flav on bacterial growth dynamics at different concentrations. a–*S*. *aureus* (MIC– 0.24 μg/mL); b–*B*. *subtilis* (MIC– 0.24 μg/mL); c–*E*. *coli* (MIC– 3.9 μg/mL); d–*K*. *pneumoniae* (MIC– 3.9 μg/mL).

The previously determined MIC’s values were used as reference to evaluate the effect of **ClCl-flav** on bacterial growth dynamics. Different concentrations of **ClCl-flav** significantly inhibited the development of all tested microorganisms during the exponential phase in MHB medium as the growth curves depicted in Fig 2 show. The dynamics also revealed that the bacterial growth was progressively inhibited by increasing the concentrations of **ClCl-flav** in the culture medium. Our compound caused a significant growth delay of *S*. *aureus* and *B*. *subtilis* (up to 9 h) at a concentration equivalent to MIC (0.24 μg/mL) compared to control. Longer delays (up to 12 hours) were evidenced when *E*. *coli* and *K*. *pneumoniae* cells were exposed to **ClCl-flav** at MIC (3.9 μg/mL)–Fig 2c and 2d. Concentrations equivalent to 2 × MIC induced a bacteriostatic effect recorded for all tested bacteria for more than 12 hours.

#### Time—Kill studies

**Fig 3:**
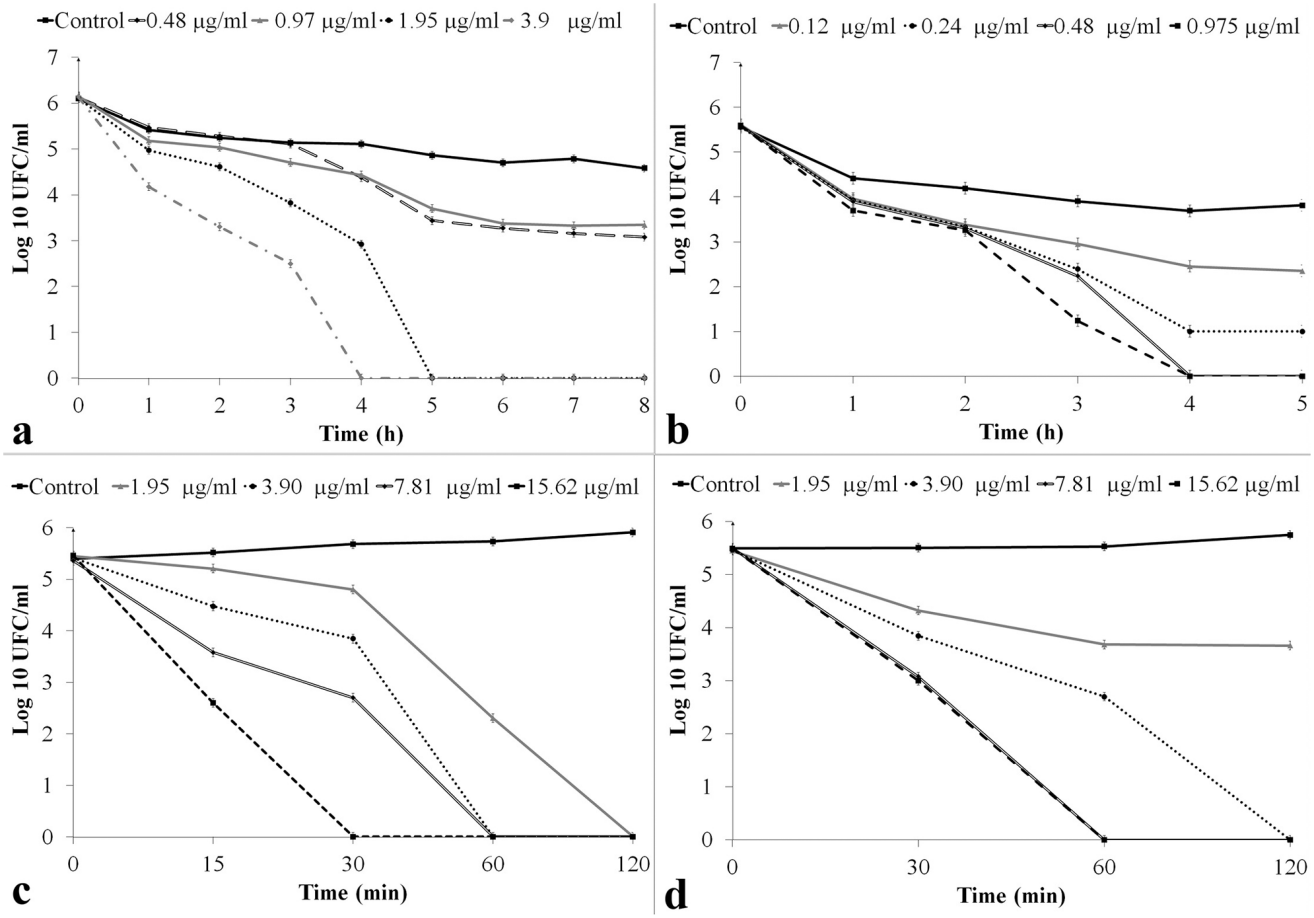
Time-kill curves after treatment with ClCl-flav at different concentrations. a–*S*. *aureus*; b–*B*. *subtilis*; c–*E*. *coli*; d–*K*. *pneumoniae*. Each symbol indicates the means for at least three replicates. Bars represent confidence interval (P < 0.05).

**ClCl-flav** showed a bactericidal potential against all tested bacterial strains (Fig 3). A reduction of cell viability with more than 3-log_10_ was recorded for *S*. *aureus* at a concentration of 1.95 μg/mL over 4 hours (Fig 3a); at two times the MIC, **ClCl-flav** induced the same bactericidal effect for *B*. *subtilis* cells within 3 hours (Fig 3b). In contrast, treatment of Gram negative bacteria with concentrations equivalent to two times the MIC resulted in reduction of viable cells with more than 5-log_10_ in only 60 minutes (Fig 3c and 3d). Also, we must emphasize that no regrowth occurred after 24 hours of **ClCl-flav** exposure for all tested bacteria at concentrations as low as 0.24 μg/mL (*B*. *subtilis*) and 7.81 μg/mL (*E*. *coli*).

### ClCl-flav impede the bacterial biofilm formation

**Fig 4:**
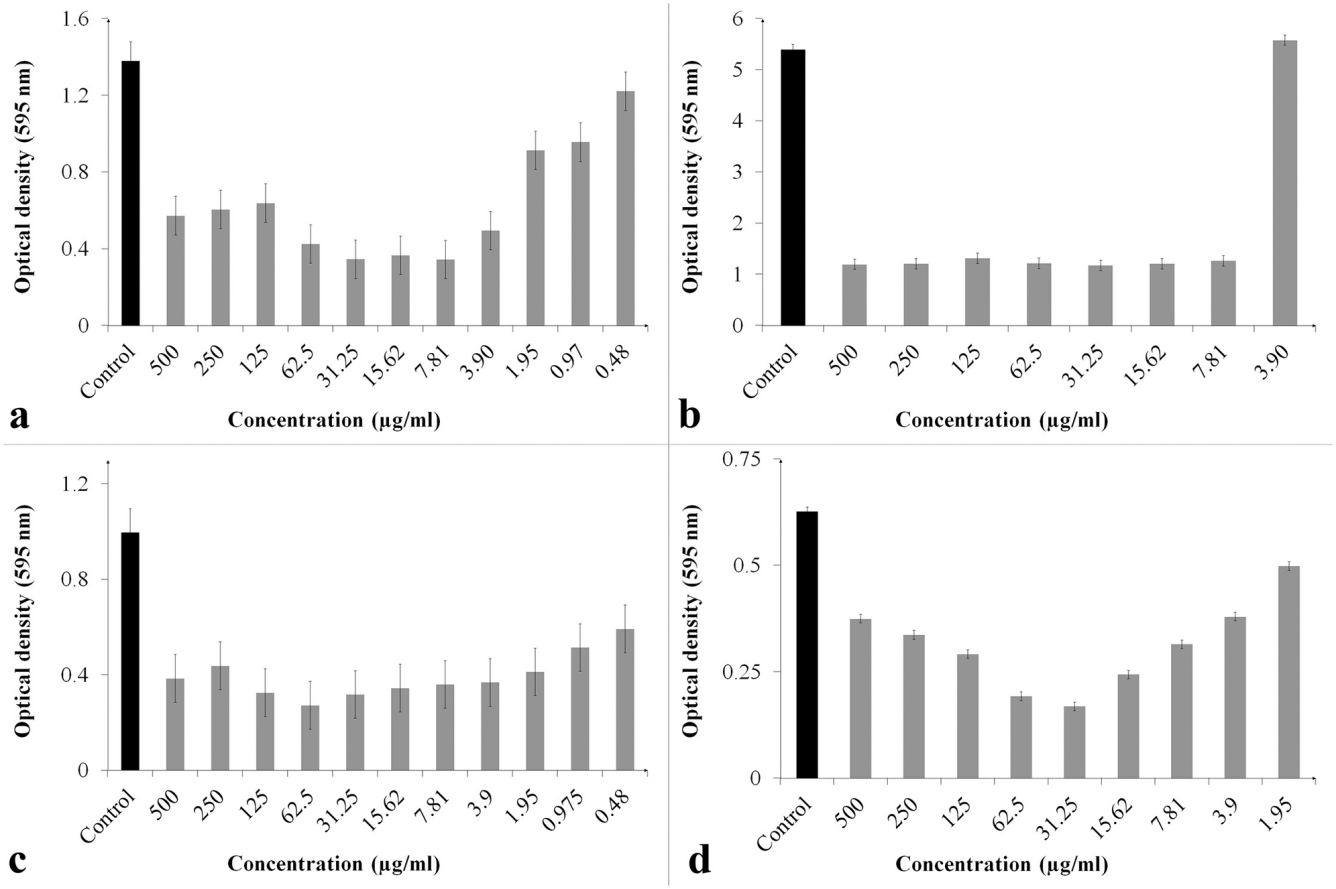
Comparative *in vitro* activities of ClCl-flav against bacterial biofilms at different concentrations. a–*S*. *aureus*; b–*B*. *subtilis*; c–*E*. *coli*; d–*K*. *pneumoniae*. Values are means of at least three replicates. Bars represent confidence interval (P < 0.05).

Our results showed that **ClCl-flav** had a significant inhibitory effect on biofilm formation during 24 hours in the case of all tested strains, as it can be seen in Fig 4. The lowest concentrations with significant anti-biofilm activity were 0.97 μg/mL for the Gram positive bacteria and 0.48 μg/mL for Gram negative bacteria. Interestingly, the lowest concentration that significantly inhibited biofilm formation by *S*. *aureus* was 0.97 μg/mL, compared to *B*. *subtilis*—7.81 μg/mL, although the minimum inhibitory concentration values were the same for the two bacteria. Also, it should be noted that despite different OD595 values depicted in Fig 4a and 4d, **ClCl-flav** induced at its most efficient concentrations a reduction of the biofilm formation with approx. 75% compared to the control for all tested bacterial strains.

### ClCl-flav impairs the cellular membrane integrity

Further experiments were conducted in order to assess the **ClCl-flav** mechanism of action against *S*. *aureus* and *E*. *coli*.

**Fig 5:**
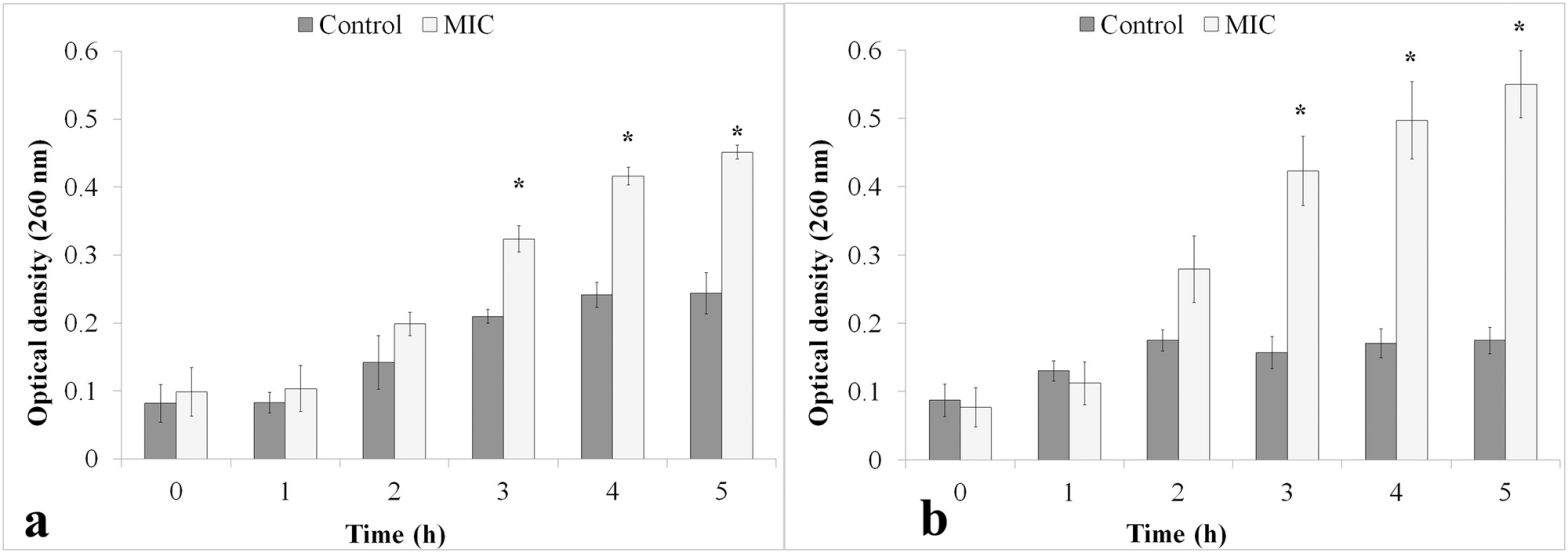
The efflux of 260 nm-absorbing materials from *S*. *aureus* (a) and *E*. *coli* (b) cells after ClCl-flav exposure. Results are presented as mean (n = 3) ± S.E.M. Asterisk denotes a significant difference (*P < 0.05) vs. Control.

**ClCl-flav** at the MIC induced leakage of 260 nm absorbing intracellular compounds during the 5 hour treatment, the recorded effect being time dependent. The OD_260_s of filtrates from treated cells suspensions were significantly higher compared to control only after 3 hours of **ClCl-flav** exposure, as it can be seen in Fig 5. The recorded loss of intracellular compounds was more pronounced for *E*. *coli* treated cells.

**Fig 6:**
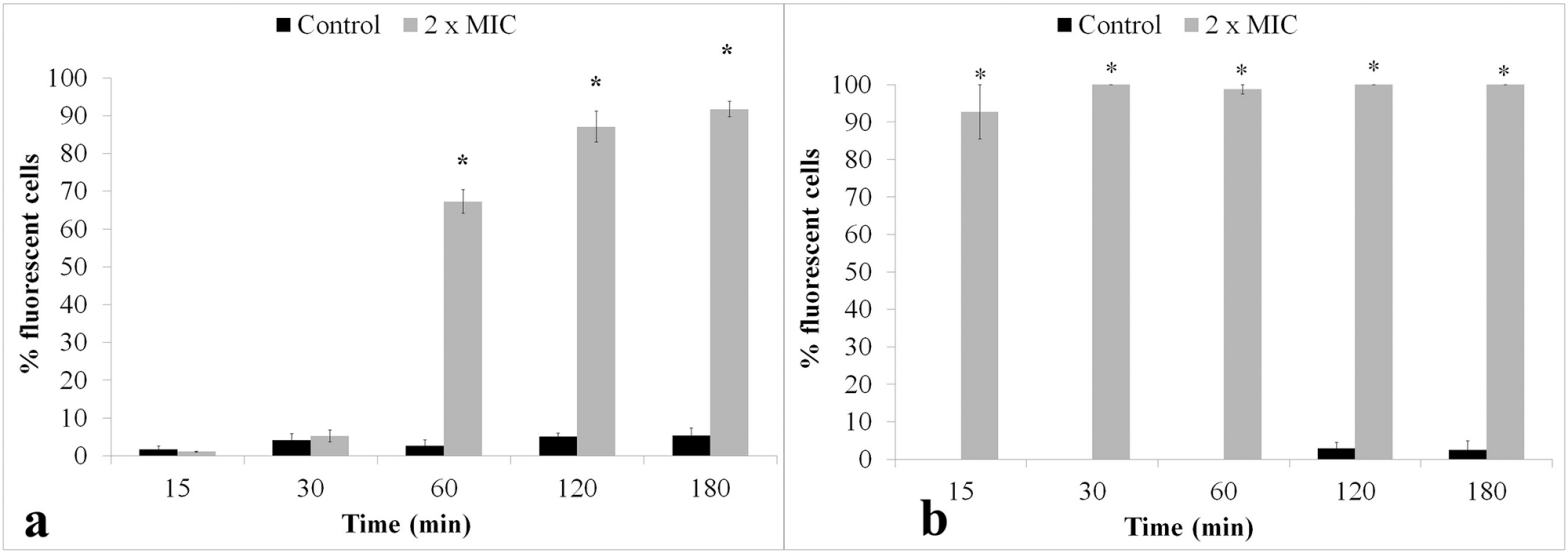
Effect of the ClCl-flav exposure on membrane permeability of exponential-phase cells to acridine orange/propidium iodide (a—*S*. *aureus*) and ethidium bromide (b—*E*. *coli*). Values are mean ± S.E.M. Asterisk denotes a significant difference (*P < 0.05) vs. Control.

Penetration of PI or EB into dead or injured *S*. *aureus* and *E*. *coli* exponential-phase cells exposed to a concentration of **ClCl-flav** equivalent to 2 × MIC was visualized using fluorescence microscopy. The inability of PI or EB to penetrate viable cells with intact cytoplasmic or plasma membranes [19] was confirmed by the low levels of uptake observed in control cells throughout the entire incubation period (Fig 6). On the contrary, exposing cell suspensions of *S*. *aureus* and *E*. *coli* to **ClCl-flav** significantly increased cell permeability to fluorescent dyes, compared to control. High percentages of fluorescent cells (92.75%) were recorded for *E*. *coli* at a concentration of 7.81 μg/mL after only 15 min of **ClCl-flav** treatment. Percentages of 100% were recorded during the whole experiment starting with 30 min after initial exposure (Fig 6b). Lower percentages were obtained for *S*. *aureus* at 0.48 μg/mL **ClCl-flav**, the recorded differences between treated cells and control being statistically significant only after 60 min of exposure (Fig 6a).

**Fig 7:**
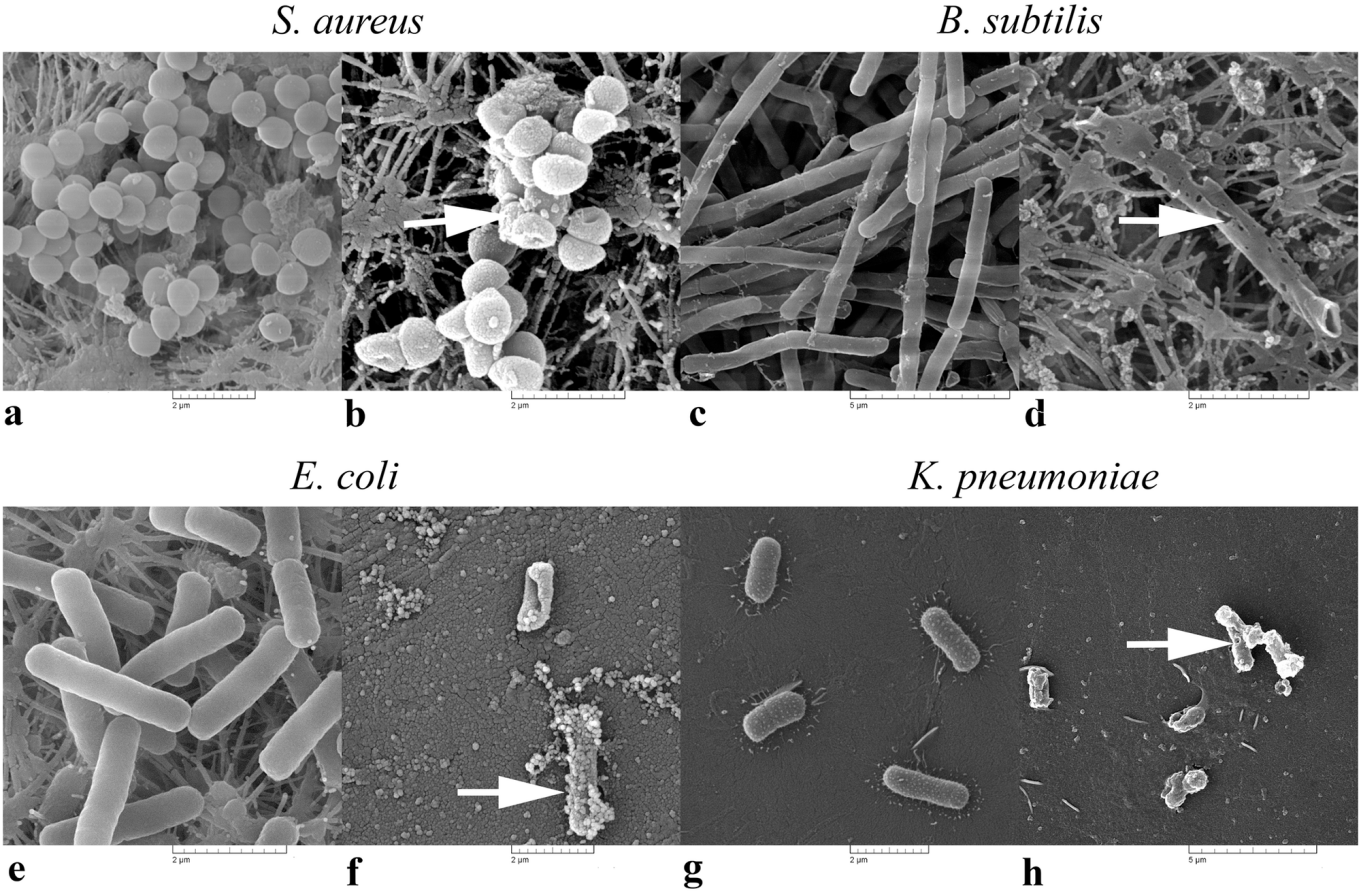
SEM photomicrographs showing the effects of ClCl-flav on bacterial cell morphology: a, c, e, g—controls; b, d, f, h—ClCl-flav exposed cells (2 × MIC). Arrows indicate irreversible morphological damage of treated bacterial cells. These scanning electron photomicrographs are representative of a typical result.

The SEM analysis clearly showed that **ClCl-flav** exposure resulted in considerable morphological damage of all treated bacterial cells when compared to the control cells (Fig 7). The cell structures appeared to be empty of content and the remains were flaccid. Smaller cell structures were observed for both Gram positive and Gram negative bacteria. For example the average cell length of treated *E*. *coli* cells was significantly lower (1.49 ± 0.04 μm) compared to the control cells (2.25 ± 0.08 μm). Also, the average cell diameter of treated *S*. *aureus* cells (0.71 ± 0.004 μm) was significantly lower compared to the control cells (0.87 ± 0.012 μm). Although the SEM samples were not prepared in a quantitative manner, the number of cells retained on the filter was lower in the cell suspension treated with **ClCl-flav** compared to control as resulted from the SEM observations.

### Cytotoxicity assessment

**Fig 8:**
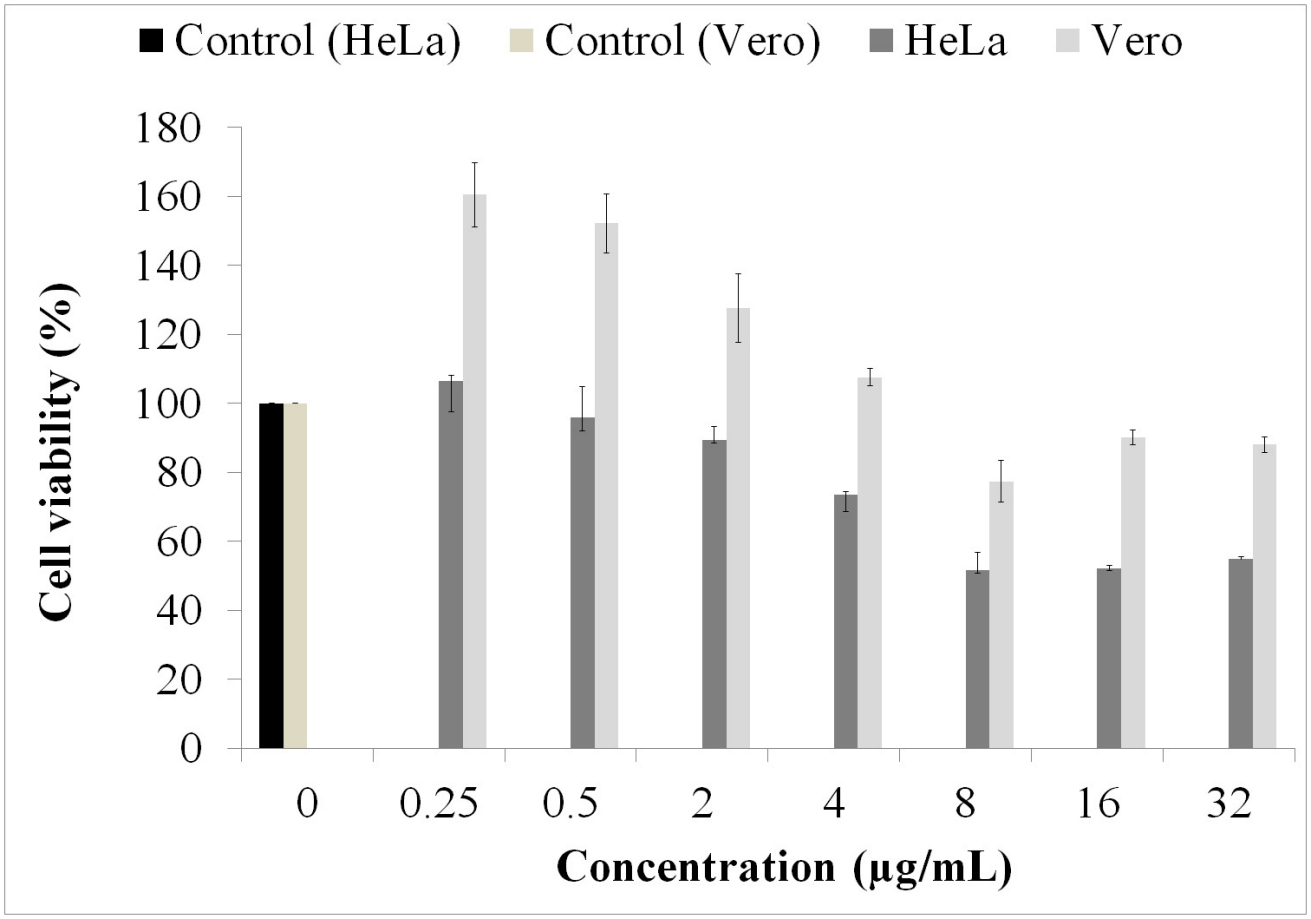
Cytotoxicity assessment of HeLa and Vero cells incubated with ClCl-flav at different concentrations. All experiments were performed in at least triplicate.

Despite the strong antibacterial activity displayed by ClCl-flav, no cytotoxic effect against Vero cells up to a concentration of 4 μg/mL and a very low cytotoxicity at higher concentration (32 μg/mL) were recorded (Fig 8). Also a significant proliferation effect was evidenced at a concentration range between 0.25–4 μg/mL.

No cytotoxic impact on HeLa cells was recorded up to 2 μg/mL while higher concentrations (4–32 μg/mL) reduced significantly the percentage of viable cells (up to 54.81%).

## Discussions

The biological activity of natural flavonoids has been the subject of many studies published before. However, the use of synthetic flavonoids as antimicrobial agents is less exploited. In addition, their mechanism of action is still unclear and often misinterpreted [7]. In this context we report here the potent antibacterial properties of a novel tricyclic synthetic flavonoid named **ClCl-flav**. Our results showed that all tested bacterial strains were susceptible to **ClCl-flav**, with MICs as low as 0.24 μg/mL against Gram positive bacteria and 3.9 μg/mL against Gram negative bacteria. Considering that compounds with MIC values less than 10 μg/mL are regarded as very interesting [7], our results suggest that **ClCl-flav** possesses high *in vitro* antibacterial activity. Moreover, our compound displayed a stronger antimicrobial activity, up to 72-fold more active against Gram positive bacteria and up to 32-fold against Gram negative bacteria when compared to other synthetic flavonoids such as 2´,4´,3-trihydroxychalcone [20], 3-*O*-alkyl-(+)-catechin derivatives [21], 7-*O*-modified genistein derivatives [22], etc. We must also emphasize that **ClCl-flav** was more effective compared to panduratin A [23] and isobavachalcone [4]–two of the most potent antibacterial natural flavonoids tested in recent years [7].

The higher sensitivity of the Gram positive bacteria to **ClCl-flav** compared to the Gram negative ones is most likely attributed to the different cell-wall structure. It is well known that the cell wall of Gram positive bacteria contains a considerable amount of peptidoglycan when compared to Gram negative bacteria. This translates to a large number of nucleophilic centers that are likely to interact with the positive carbon atom of the 1,3-dithiolium ring of **ClCl-flav** and therefore, better activity against *S*. *aureus* and *B*. *subtilis* compared to *E*. *coli* and *K*. *pneumoniae* [8].

The same strong antibacterial effect was also revealed by the growth dynamics in liquid medium. The inhibition effect was dose-dependent, increasing concentrations of **ClCl-flav** progressively inhibited the growth of all tested bacteria. Significant growth delays were recorded at concentrations equivalent to MIC, while 2 × MIC concentrations induced a bacteriostatic effect for more than 12 hours. Moreover, **ClCl-flav** exposure induced a bactericidal effect as it was evidenced by the time-kill assays. Total kill (no viable cells recovered) was achieved at different concentrations: 0.24 μg/mL (*B*. *subtilis*), 0.97 μg/mL (*S*. *aureus*), 7.81 μg/mL (*E*. *coli*) and 15.62 μg/mL (*K*. *pneumoniae*) with no regrowth after 24 hours. At twice the MIC, all *E*. *coli* and *K*. *pneumoniae* cells were killed within one hour. A comparative literature study revealed that **ClCl-flav** has a higher bactericidal activity, up to 100-fold more active against Gram positive bacteria and up to 8-fold against Gram negative bacteria compared to most of the reported synthetic flavonoids [24–26].

Besides the bacterial killing activity, **ClCl-flav** also impeded the biofilm formation at concentrations as low as 0.97 μg/mL recorded for the Gram positive bacteria and 0.48 μg/mL for Gram negative bacteria. Also it should be noted that **ClCl-flav** didn’t induced a dose-dependent inhibitory effect on biofilm formation, with lower concentrations leading to a stronger anti-biofilm activity effect when compared to the highest concentrations used (e.g. 500, 250 or 125 μg/mL). Irreversible inhibition of bacterial growth as well as anti-biofilm activity, are desirable properties to prevent bacterial colonization of different surfaces such as medical devices, where bacterial killing activity is required [27]. Therefore our experimental results opened the possibility of using **ClCl-flav** for the development of an effective surface sterilizer or as a reliable solution for food packaging or biomaterial industry.

**ClCl-flav** exposure of *E*. *coli* and *S*. *aureus* cells also induced an increased uptake of EB or PI respectively, to which the cell membrane is normally impermeable [19]. Such observations indicate that **ClCl-flav** impaired the cell membrane and rendered the intracellular nucleic acids accessible to PI or EB. The percentages of fluorescent cells were higher at the beginning of incubation for *E*. *coli* compared to *S*. *aureus*, but this could be rather related to the cell interaction with PI used as fluorescent marker since *S*. *aureus* was more sensitive to **ClCl-flav** compared to *E*. *coli*.

Bacterial cells suspensions treated with **ClCl-flav** lost significant amounts of 260-nm absorbing intracellular compounds, suggesting that nucleic acids were lost due to a damaged cytoplasmic membrane. The leakage of cytoplasmic material is considered indicative of gross and irreversible damage to the cytoplasmic membrane and is commonly quantified by the loss of intracellular material that absorb at wavelengths of 260-nm (nucleic acids) [14]. Similar to **ClCl-flav** many antimicrobial compounds that act on the bacterial cytoplasmic membrane induced the loss of 260-nm absorbing material [28, 29].

The results presented so far show that **ClCl-flav** irreversibly modifies the integrity of the cell membrane, allowing the penetration of PI or EB and the leakage of nucleic acids from the cell. These effects occur after only 15 minutes for *E*. *coli* and 60 min for *S*. *aureus* (Fig 6). Probably at this time **ClCl-flav** do not affect significantly the viability of all exposed cells, being a known fact that not all fluorescent cells are also dead [30]. After 2 hours of **ClCl-flav** exposure, the integrity of the membrane is irreversibly impaired for both Gram-positive and Gram-negative bacteria, which leads to the leakage of nucleic acids from the damaged cells and affects the viability as showed by the results of the bacterial killing assay (Fig 3).

The hypothesis that **ClCl-flav** targets the cell membrane is also supported by the SEM analysis. The photomicrographs showed major cell damage and loss of cytoplasmic material due to cell lysis. After loss of contents, cells treated with **ClCl-flav** collapsed and became smaller. This enabled them to pass more easily through the pores of the filter when compared to untreated control cells. Those observations sustain the hypothesis of membrane disruption induced by **ClCl-flav** treatment. The SEM images also showed that the number of disintegrated cells at the same exposure time (6 hours) and concentration (equivalent to 2 × MIC) were higher in the case of *E*. *coli* compared to *S*. *aureus*. This could be related to the differences between Gram positive and Gram negative bacteria concerning the ultrastructure and chemical composition of the bacterial cell wall, marked by the absence or presence of an outer lipid membrane [31]. The Gram negative bacteria are more rapidly destroyed due to the **ClCl-flav** attack on both outer and plasma membranes, which leads to rapid cell disintegration. In the case of Gram positive bacteria, even if the membrane is irreversible damaged, the cells are not so rapidly disintegrated due to the thick peptidoglycan layer responsible for maintaining the cell architecture for a longer time.

Practical applications of **ClCl-flav** as a potential antibacterial agent depend on its cytotoxicity. Our results showed no toxicity against Vero cells at effective concentrations against tested bacteria (4 μg/mL) and a low toxicity for higher doses (up to 32 μg/mL). Moreover, a higher number of viable cells was recorded (expression of an increased rate of proliferation) as compared to the control group in the dose interval of 0.25–4 μg/mL. Overall, the tested compound exerted a biphasic effect on normal Vero cells in terms of proliferation and cytotoxicity. Similar effects of polyphenolic compounds were also signaled on other cell lines, the biphasic behavior being dose-dependent [32].

Increased uptake of fluorescent dyes, significant loss of cytoplasmic material and cell lysis suggests that the cell membranes were severely compromised due to **ClCl-flav** exposure. All data point out to a membrane-based mechanism of action. Most likely, this is the result of several different modes of action that may include the chemical and or electrostatic interactions between the tricyclic flavonoid and nucleophilic sites of the outer and plasma membranes (dicarboxylic aminoacids from porins, phosphate groups etc.).

In conclusion, our observations suggests that **ClCl-flav** is an antimicrobial agent with potent *in vitro* antibacterial properties against Gram positive and Gram negative representative bacteria and with no or very low cytotoxicity against Vero cells. The antimicrobial activity results from its ability to damage the cell membrane structures and to affect the permeability. Our results strongly suggest that **ClCl-flav** should undergo further testing to assess its potential to be used as an antimicrobial drug or as a sterilizer. Further studies are necessary to elucidate its antibacterial activities *in vivo*.
